# From concrete to canopy: Illuminating moth biodiversity in New York City’s urban jungle

**DOI:** 10.1371/journal.pone.0342856

**Published:** 2026-05-12

**Authors:** Shira Linsk, Anna Thonis, Kristin M. Winchell

**Affiliations:** Department of Biology, New York University, New York, New York, United States of America; Zoological Survey of India, INDIA

## Abstract

Moths (Lepidoptera) are sensitive to anthropogenic threats and serve as valuable bioindicators. Despite the remarkable diversity and abundance of Lepidoptera globally, there is a lack of information on how moth species are impacted by urbanization. Notably, very little is known about moths in the most populus city of the United States, New York City, where pervasive urban pollutants, artificial light at night, land cover change, and habitat fragmentation are severe. We examined the effects of urbanization on moth biodiversity in New York City, with a focus on green spaces. We used citizen science records from *iNaturalist* and complemented these data with ground sampling at twelve locations across six parks at night. While the *iNaturalist* dataset is comprehensive both spatially and temporally, it failed to detect some species we observed on the ground. However, the scope of the field survey dataset is limited in geographical breadth and seasonal coverage. Overall, we found a negative relationship between greater urbanization and moth diversity, with community similarity related to environmental similarity. Our results found greater biodiversity with less light at night and less urban development, and more deciduous tree cover and more open land. Our structural equation model reveals additional insight: although we detected a strong direct negative effect of developed land on moth diversity, urbanization also negatively impacts diversity via indirect effects of reducing open space and deciduous tree cover. Developed open space alone does not directly affect diversity but may positively impact diversity through its covariance with vegetation cover. These findings support the importance of mitigating artificial light at night in urban green spaces and maintaining urban vegetation to ensure nocturnal Lepidoptera can persist in rapidly urbanizing landscapes.

## Introduction

Urbanization is linked to declines in biodiversity through biotic homogenization and species extirpation [1,2]. These impacts are seen across the tree of life, with steep declines observed across insect orders [3,4], resulting in local extinctions and reduced insect diversity [5,6]. Much of this work has focused on bees and butterflies, revealing decreased diversity and abundance with increased urbanization [5–8]. Urbanization presents many challenges for insects, including the urban heat island effect, habitat fragmentation, air pollution, artificial light, impervious surfaces, and exotic plants [9]. Habitat fragmentation and landscape maintenance are major contributors to pollinator decline, including in Lepidoptera [10]. However, alongside these challenges come opportunities in terms of anthropogenic resources that may be exploited. This is perhaps most obvious when considering pest species like cockroaches, flies, and carpet beetles that persist in cities despite the threats of pest control [11]. Yet even non-pest species may find suitable habitat in cities that allow them to persist, albeit in many cases not as successfully as in non-urban environments. For example, urban plantings may generate habitat and resources that support invertebrate diversity, including Hymenoptera and spiders [12–15].

Although these diversity trends have been documented before, relatively little research on urban invertebrates has focused on moths, and there is a notable bias toward butterflies and other diurnal Lepidoptera [6,16,17]. This is perhaps due to the challenges associated with observing moths at night (e.g., low visibility, general safety considerations) and the minimal public engagement surrounding them compared to butterflies [18]. Some recent studies on moths have found decreasing populations with increased artificial light at night [19–21]. Additionally, anthropogenic environments generate intense selection pressures and filtering processes that may favor the survival of only a few species, such as heat-tolerant moths [22] or those with larger body sizes [23]. Indeed, studies of industrial melanism in moths represent some of the first examples of rapid evolutionary change in cities [24–26].

Understanding the effects of urbanization on moths is important because of the crucial roles they play in ecosystem services (e.g., pollination, food, and nutrient cycling). Moths are fundamental to ecosystem health and stability and serve as bioindicators [21,27] since they respond quickly to environmental change [19,28,29]. There are approximately 150,000 known species of moths globally [30], and hundreds of new species are described annually [31]. Yet, we know very little about which species persist in urban landscapes. For conservation initiatives to be successful in these spaces, we must first take stock of the species that reside there and relate patterns of diversity to urban stressors.

We investigate patterns of moth diversity in New York City, where artificial light at night, air pollution, and habitat fragmentation are extreme. We hypothesize that landscape-scale features influence species diversity and predict that developed land use negatively impacts moth diversity. In addition, we hypothesize that habitat-scale features such as microclimate, air pollution, and light at night will be significant predictors of moth biodiversity, with greater air pollution, more light at night, and increased temperatures associated with decreased diversity. By connecting moth diversity to urban habitat features, we aim to improve our understanding of the ecological impacts of urbanization on nocturnal Lepidoptera species in one of the largest urban centers globally.

## Materials and methods

### *iNaturalist* data

**Fig 1:**
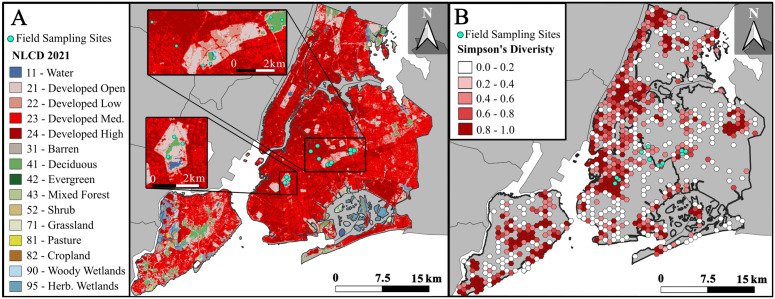
Study sites and moth diversity patterns across New York City. **(A)** On-the-ground sampling locations for 12 locations in Brooklyn and Queens, New York City, USA, where we sampled Lepidoptera, and National Land Cover (NLCD) data for New York City. Insets represent magnified views of study sites. **(B)** Species diversity in 1 km hexagonal bins derived from *iNaturalist* data.

We downloaded 31,793 Research-Grade observations of the Order Lepidoptera using a polygon spatial filter around all five New York City boroughs. Research grade observations refer to records for which a minimum of two members of the *iNaturalist* community agree on the species-level identification. We manually removed butterfly Families (i.e., Papilionidae, Nymphalidae, Pieridae, Lycaenidae, Hesperiidae), resulting in 12,956 moth observations. We imported records into QGIS (ver. 3.34.3-Prizren) [32] and used the New York City administrative boundary layer [33] to reduce this dataset to 10,740 records located within New York City boundaries. We used the plugin *Density Analysis* [34] to quantify observation density using a grid of 1 km hexagonal cells across the region (Fig 1). We excluded hexagonal zones (33 zones) with undefined landscape types or >70% water cover (according to the National Land Cover Database “NLCD” [35]), to ensure the zones had relevant habitat data for terrestrial species, retaining 757 zones. We filtered the *iNaturalist* records to include observations from 2000 onwards to better capture moth diversity over time, rather than at one specific point in time. This cutoff was chosen based on the possible unreliability of metadata from older observations for which date and location would have to be manually entered (i.e., prior to digital imaging) years after the observation was made. Additionally, we removed moth observations that we ourselves contributed during the course of the surveys to avoid double-counting records in our combined analyses. This resulted in a total of 9,374 records.

### Field site selection

To complement our *iNaturalist* dataset and provide field-based comparisons with any observed trends, we identified 12 on-the-ground sampling locations within six greenspaces across Brooklyn and Queens, New York, that reflect variation in urban-associated land cover and habitat features encountered across the city (Fig 1; S1 Table). In larger parks (Highland, Forest, Prospect Parks) we identified multiple locations at least 400m apart representing different surrounding land-use compositions and treated each sampling location as unique rather than a replicate of the larger park. We visually assessed satellite imagery to identify green spaces and then chose sampling locations within each space spanning a gradient in light and air pollution based on on-the-ground measurements (rather than remote-sensed) to capture variation in these variables at a finer, site-level scale.

### Field sampling

In accordance with the requirements of the New York City Parks Department, we acquired a permit to sample across New York’s public parks. We surveyed each sampling location during a single sampling period for one hour shortly after dusk. The exact time at which sampling occurred varied throughout our survey timeframe as sundown began later into the evening as the season progressed. We restricted the temporal sampling period to a two-month period (58 days) to minimize seasonal variation in species detected across sampling locations, with locations in the large parks sampled on different days. We constructed a light trap using a suspended 5 m^2^ white sheet directly illuminated with two flood lights (6000 Kelvin, visible light spectrum) and one ultraviolet LED light (385–395 nm) (as in [36]). Moths are easily sampled with light traps, allowing for robust estimates of species richness and abundance [28]. Each survey consisted of two people photographing all moths observed landing on or in the immediate vicinity (on illuminated ground within 2m) of the light trap. We identified species from the photographs and counted the total number of individuals per species encountered during each sampling event. In addition, for each sampling event, we measured the following site-level variables: air pollution (PM 2.5; Temtop M2000C), light at night (lux; EXTECH Light Meter LT300), temperature, humidity, and wind speed (Kestrel 3000).

### Landscape analysis

We mapped *iNaturalist* records and on-the-ground sampling locations using QGIS. We extracted zonal statistics of NLCD land cover classifications [35] within each 1 km hexagonal zone for the *iNaturalist* dataset. Our dataset included NLCD classifications (defined by modified Anderson Level II classification [37]) as follows: Deciduous Forest (trees > 5m tall dominate vegetation with >75% of trees seasonally losing foliage), Mixed Forest (trees > 5m tall dominate vegetation with both deciduous and evergreen species up to 75% representation), Grassland (unmanaged lands dominated by graminoid or herbaceous vegetation), Pasture/Hay (vegetation planted for livestock grazing or crop production), Developed Open Space (a mixture of buildings and vegetation, mainly lawns and parks, with <20% impervious surface), Developed Low-Intensity (a mixture of buildings and vegetation with impervious surfaces 20–49% of cover), Developed Medium-Intensity (a mixture of buildings and vegetation with impervious surfaces 50–79% of cover), Developed High-Intensity (dense human settlements with 80–100% impervious surface cover), and Water (open water with <25% soil or vegetation cover). For on-the-ground sampling locations, we used a 150 m buffer around each sampling point to capture the immediate landscape characteristics of each site and at a spatial scale relevant to Lepidoptera community structure (100-200 m) [38]. We calculated the percentage of each land cover class for hexagonal zones and buffered areas. We then transformed these compositional proportions (fractions of area that are water, forest, etc.) from both *iNaturalist* and using an Isometric Log-Ratio (ILR) transform [39] using the package *compositions* [40] and *zCompositions* [41]. We then summarized the transformed land cover classifications with principal component analysis (PCA) using the function ‘prcomp’ in base R *stats* [42]. We back transformed the ILR basis to inspect the loadings of each land cover class for each principal component (PC). We then visualized results with packages *factoextra* [43] and *corrplot* [44].

### Statistical analyses

Statistical analyses were conducted using R studio (34, ver. 2023.12.0.369) and R (ver. 4.3.2) [45]. For all linear models, we evaluated multicollinearity with the ‘VIF’ function in the package *car* [46] assessed statistical significance using t-tests for individual coefficients. We used the function ‘step’ (package *stats*) for model simplification (as noted below), which evaluated model fit based on Akaike Information Criterion (AIC) and residual deviance [47].

We summarized species diversity with Simpson’s Diversity Index (Simpson’s D), which considers both species richness and evenness [48]. Simpson’s Diversity Index is robust in urban settings as it is not sensitive to rare taxa that may be under-sampled or absent in disturbed habitats where common generalist species might dominate [22]. Additionally, this index can overcome issues of small sample size and spatial biases [49]. We calculated Simpson’s D (1-D) using species-level identification for each *iNaturalist* hex bin and each on-the-ground sampling location in R using the function ‘diversity’ in the package *vegan* [50].

We conducted three analyses using the *iNaturalist* data*.* First, we used multiple matrix regression (MRM) to analyze the similarity of community composition with respect to environmental similarity across sites This entailed generating a distance matrix (Euclidean Distance) of species composition similarity across hexagonal zones, as well as for the environment (percentages of all NLCD land cover classes). We used the function ‘dist’ in the R base *stats* for all distance matrix analyses [42] and performed matrix regression using the function ‘MRM’ in R package *ecodist* [51]. Second, we used linear regression to analyze the relationship between moth diversity (Simpson’s D) and landscape-level (remotely sensed) habitat features. Explanatory variables in the model included NLCD categories (as a proportion of hex-bin area): deciduous tree cover, developed land cover (high-, medium-, low-intensity), pasture, developed open land, shrub, and grassland. We simplified this model using stepwise model simplification (backwards and forwards) based on AIC using the function ‘step’ in R base *stats*.

In addition, we developed a structural equation model (SEM) using *iNaturalist* observations to examine relationships between moth diversity, vegetation (deciduous forest, grassland, pasture, shrub), developed land (high-, medium-, low-intensity), and developed open land cover. SEMs analyze multiple relationships at the same time while considering 3 latent factors (i.e., unobserved relationships): (1) Vegetation (deciduous land cover, grassland, pasture, and shrub), (2) Developed land (Developed High-, Medium-, and Low-Intensity) and (3) Developed open space. We specified the model to reflect the hypothesis that different components of the urban landscape (i.e., vegetation, developed land, and open space) each exert direct effects on moth diversity, and that these land cover types may covary due to the spatial structure of urban environments (e.g., lower vegetation cover in highly developed areas). Latent variables were used to group related land cover types, reducing collinearity and capturing broader ecological gradients. We fit our SEM using maximum likelihood with the ‘sem’ function in the *lavaan* R package (ver. 0.6–19) [52]. Model fit was assessed using Chi-squared (χ²), Comparative Fit Index (CFI), Tucker-Lewis Index (TLI), and Root Mean Square Error of Approximation (RMSEA). To account for shared variance between variables, we specified covariances between vegetation cover and development intensity, development intensity and developed open space, and between several vegetation variables, including between deciduous and grassland, and between grassland and shrub. We used the z-statistic to assess the significance of each path coefficient in the SEM and visualized observed relationships with the function ‘semCors’ in the R package *semPlot* [53].

We conducted two additional analyses using our on-the-ground field sampling data. We again analyzed the similarity of community composition concerning environmental similarity across sites using MRM, removing the two sites where no moths were sampled. As with the *iNaturalist* MRM analysis, this entailed generating a distance matrix of species composition similarity across sampling sites, and for the environment: percentages of all NLCD land cover classes, as well as PM2.5, lux, temperature, humidity, and wind speed measured on the ground at time of sampling. Second, we used linear regression to analyze the relationship between moth diversity and site-level habitat features. We also included sampling date as a numerical covariate to better account for seasonal effects over the sampling period. We did not include remotely sensed land cover data in this model because the resolution of these data and our sample size resulted in low statistical power (overparameterization) and multicollinearity when these variables were included. Explanatory variables used in our model included temperature, pollution (PM 2.5), and light intensity (lux) measured during on-the-ground surveys. We simplified this model using stepwise model simplification (backwards and forwards). Lastly, we combined *iNaturalist* data with our on-the-ground survey data to evaluate the relationship between diversity and urbanization more broadly. Specifically, we analyzed a linear regression of diversity by the first four principal components in our land cover PCA, with no interactions or model simplification.

## Results

### Urban environmental variation

**Fig 2:**
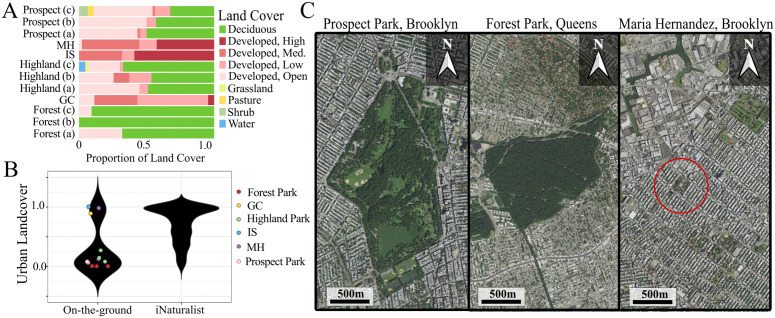
Land cover composition, principal components analysis, and site imagery of sampling locations. **(A)** Stacked bar plot of NLCD land cover composition at on-the-ground sampling sites. Colors represent the proportion of each land cover type in 150 m buffers around each sampling location. **(B)** Violin plots represent the sum of urban NLCD land cover proportions (developed land of high, medium, and low intensity) for our on-the-ground sampling locations and all hex-bins used with *iNaturalist* data. **(C)** Satellite images of sampling locations in Brooklyn and Queens, New York. Satellite images courtesy of the USDA, USGS The National Map: Orthoimagery.

Land cover analyses using remotely sensed data verified that our on-the-ground sampling locations spanned a broad range of urbanization representative of New York City (Fig 2A-B). Larger parks (Forest Park, Highland Park, Prospect Park) exhibited greater habitat heterogeneity and had more deciduous forest cover. Small parks (Maria Hernandez, Grover Cleveland, Irving Square), by contrast, had little to no deciduous tree cover and were composed almost entirely of developed land. Grover Cleveland Park stood out with only a small percentage of high-intensity developed land as it borders a private cemetery with considerable greenspace. These differences are clearly visible in satellite imagery: Forest Park is dominated by forest, Prospect Park has less tree cover, and Maria Hernandez is substantially smaller and consists mostly of developed land (Fig 2C).

Our PCA incorporated land cover for both *iNaturalist* records and on-the-ground sites (757 *iNaturalist* hexagonal zones, 12 on-the-ground sampling locations). The PCA captured 79.58% of the variance in the first four principal components, with PC1 capturing 30.09% of the variance. Visual inspection of the scree plot suggests that four principal components should be retained to describe variation in the dataset. The first principal component (PC1) represents urbanization with strong positive loadings for high and medium intensity developed land and strong negative loadings for developed open space and water. Principal component 2 (PC2) represents more rural conditions, with strong positive loadings for low intensity developed land and open space with strong negative loadings for grassland. Principal component 3 (PC3) represents forested conditions with strong positive loadings for deciduous land cover and strong negative loadings for high intensity developed land and water. The fourth principal component (PC4) has strong positive loadings for shrub and strong negative loadings for pasture.

### Diversity of Lepidoptera

**Fig 3:**
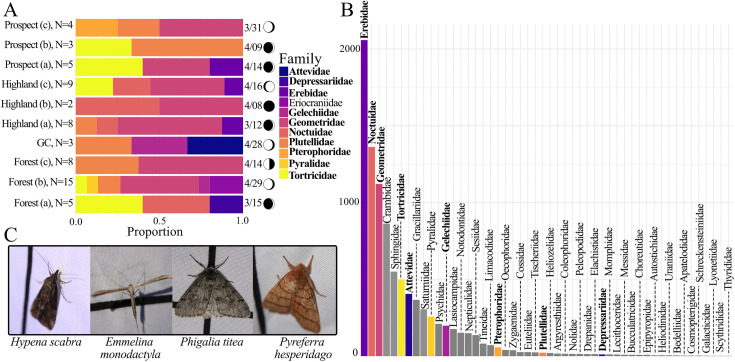
Lepidoptera family diversity from field sampling and *iNaturalist* data. **(A)** Stacked bar plot of Lepidoptera Family diversity per site for on-the-ground sampling. The left side of the plot has number of moths sampled per site **(N)**. To the right of the chart is the date each site was surveyed and the corresponding moon phase. Bolded names represent families that were observed in both our field surveys and on *iNaturalist*. **(B)** Number of observations of each Lepidoptera Family across all *iNaturalist* hex bins. Bars are colored by Families observed. Bolded family names represent observations found in both field data and *iNaturalist* datasets. **(C)** Images of some moths taken during on-the-ground sampling.

From *iNaturalist*, we extracted data from 9,374 Research Grade observations, representing 972 species, 535 genera, and 50 families (Fig 3B). In our on-the-ground surveys, we observed a total of 69 individuals, representing 11 families, 31 genera, and 33 species (Fig 3A). Our field observations were identified to species in 100% of samples using a combination of *iNaturalist* Research Grade identifications, Covell’s Field Guide [53], and the Moths and Butterflies of North America project [54–55]. We gathered suggestions from the *iNaturalist* algorithm for higher taxonomic suggestions (i.e., family or genus) and used these resources to further identify each moth to the species level. Identification to species can be challenging for many micro-moth families without internal dissections. Our on-the-ground data set was mainly comprised of either well-characterized or medium-to-larger families with distinct morphological characteristics, making identification possible (Fig 3C). However, we note that species-level identifications in the *iNaturalist* data set may contain misidentifications for micro-moth species.

To directly compare *iNaturalist* moth richness with surveyed richness, we subsetted *iNaturalist* observations to the dates of our sampling window across all years. Richness overall was higher for all the parks we surveyed when compared to the restricted *iNaturalist* sampling window. We documented 12 species in Prospect Park (compared to 10 on *iNaturalist*), 22 species in Forest Park (1 on *iNaturalist*), 19 in Highland Park (1 on *iNaturalist*), and 3 in Grover Cleveland (none on *iNaturalist*). In the two parks where no species were detected in our on-the-ground sampling (Maria Hernandez and Irving Square), there were also no observations on *iNaturalist*. Notably, even when the *iNaturalist* was temporally unrestricted we still documented higher richness in two large parks: Forest Park (*iNaturalist* total of 35) and Highland Park (*iNaturalist* total of 9). However, the temporally unrestricted dataset had markedly higher richness for Prospect Park (110 species on *iNaturalist*). Another notable finding is that we detected individuals from the family Eriocraniidae in our on-the-ground sampling, but this family was not present in the *iNaturalist* dataset. Conversely, *iNaturalist* observations documented 40 additional families not detected in our on-the-ground sampling in the temporally unrestricted dataset.

Based on our on-the-ground sampling, large parks (Forest Park, Highland Park, Prospect Park) displayed the highest levels of biodiversity (1-D: 0.44–0.88). Among them, Forest Park had a mean diversity index of 0.78 ± 0.09 (mean±sd), Highland Park had a mean diversity of 0.71 ± 0.19, and Prospect Park had a mean diversity of 0.64 ± 0.17. In contrast, the small parks (Maria Hernandez, Grover Cleveland, Irving Square) exhibited lower diversity (1-D: 0–0.66, mean±sd: 0.22 ± 0.38). No species were detected at Maria Hernandez and Irving Square in our on-the-ground sampling; since Simpson’s Diversity Index is undefined in the absence of observations, we recorded 1-D as 0 for these sites.

### Community composition

**Fig 4:**
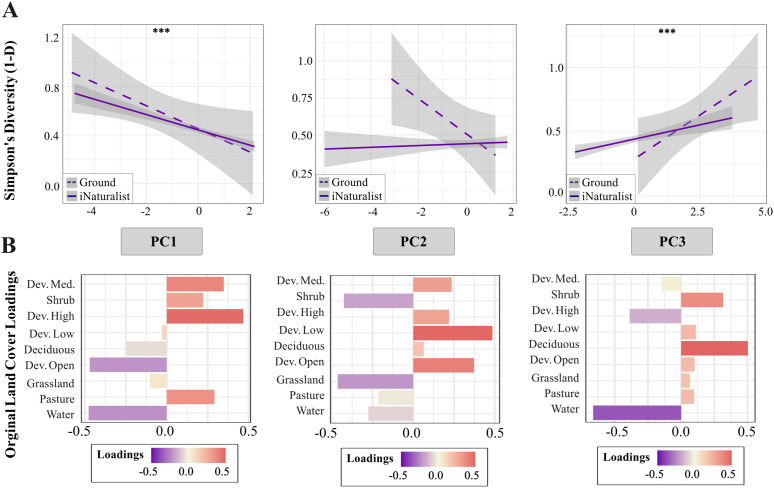
Relationships between Simpson’s Diversity Index and NLCD land cover. **(A)** Simpson’s diversity (1-D) as it relates to land cover summarized by principal components analysis (PC1, PC2, PC3), with 95% confidence intervals (shaded area). Significance levels are denoted by asterisks: p < 0.001 (***). **(B)** Land cover loadings for each principal component in the corresponding plot in part A immediately above each. PC1 represents more highly urbanized land and less open space and water. PC2 represents more low intensity developed land and open space with less herbaceous land cover. PC3 represents more deciduous forest and shrub with less highly developed land and water.

In both our *iNaturalist* data and on-the-ground sampling, locations with similar environments had similar community composition at the species level (multiple-matrix regression; on-the-ground: F = 29.442, R^2^ = 0.406, p = 0.0304; *iNaturalist*: F = 3867.86, R^2^ = 0.013, p < 0.001). Species diversity has a strong negative relationship with PC1 and a strong positive relationship with PC3 (representing increased urbanization and deciduous forest, respectively) across both datasets combined (PC1 estimate = −0.064 ± 0.008, t = −7.948, p < 0.001; PC3 estimate = 0.048 ± 0.011, t = 4.448, p < 0.001) (Fig 4). Diversity was not significantly related to PC2, representing less grassland and more low intensity developed land and open space, (t = 0.276, p = 0.783) nor with PC4, representing less pasture and more shrubbery (t = −1.894, p = 0.059).

### Relationships between environment and biodiversity

Our two linear models—one using *iNaturalist* observations with remotely sensed environmental variation and the other using on-the-ground sampling with field-measured environmental variation—revealed relationships between environmental features and biodiversity across both spatial scales. Our simplified linear regression of Simpson’s Diversity by land cover using *iNaturalist* and remotely sensed data retained five significant variables: medium-intensity developed land, low-intensity developed land, developed open land, shrubland, and deciduous tree cover. Diversity was negatively associated with medium-intensity developed land cover (estimate = −0.350 ± 0.064, t = −5.493, p < 0.001) and positively associated with deciduous tree cover (estimate = 0.465 ± 0.107, t = 4.347, p < 0.001) and developed open space (estimate = 0.375 ± 0.118, t = 3.169, p = 0.002). There was also a near-significant positive trend between diversity and low-intensity developed space (estimate = 0.268 ± 0.153, t = 1.755, p = 0.080), and although shrubland was retained in the best model, the relationship with this predictor was non-significant (t = 1.598, p = 0.110). In comparison, our simplified regression of Simpson’s Diversity for on-the-ground sampling retained one variable: Simpson’s diversity was negatively related to light at night (estimate = −0.474 ± 0.056 lux, t = −8.472, p < 0.001).

### Structural equation model

Our SEM exhibited an overall good fit to the data (CFI: 0.954, TLI: 0.914, RMSEA: 0.069). The chi-square statistic was significant (χ² = 86.993, df = 19, p < 0.001), although this is expected with larger sample sizes, and the overall fit indices suggest the model effectively captured the relationships in the data.

**Fig 5:**
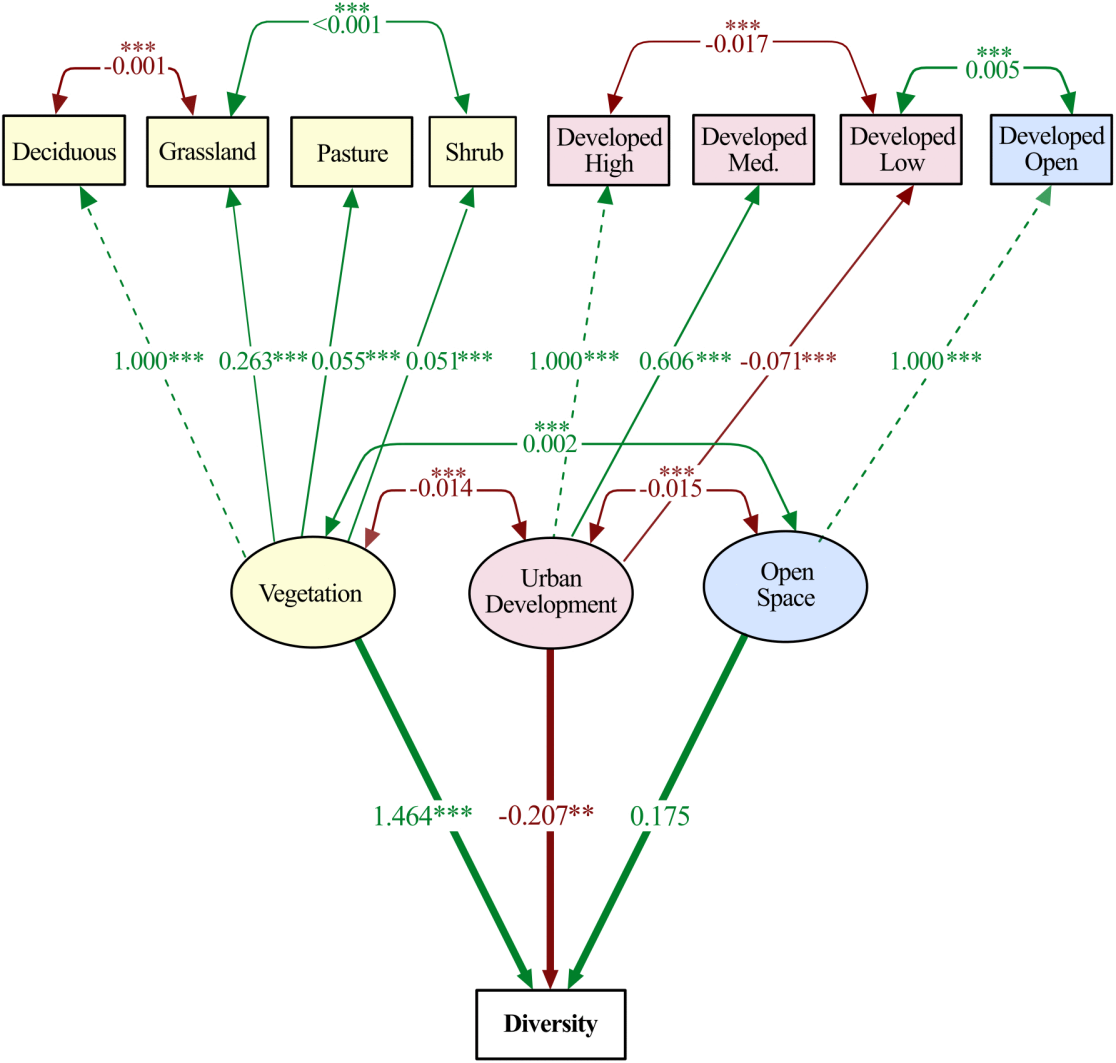
Pathways linking urban land cover to Lepidoptera diversity revealed by structural equation modeling. Relationships among predictor variables and Simpson’s diversity generated by the structural equation model using reflective syntax. The top row of land cover classifications makes up the observed variable group, while the middle row represents the 3 latent variables. The thin single-headed arrows (pointing from latent to observed) denote the loadings of each land cover. Dashed lines represent the marker variables used to scale the latent construct. The double-headed arrows represent covariance between variables while the bolded lower arrows demonstrate the structural regression paths. Significance levels are denoted by asterisks: p < 0.05 (*), p < 0.01 (**), p < 0.001 (***). Nodes represent variables included in the model, while edges indicate associations between them. Thicker lines represent stronger relationships, with positive effects shown in green and negative effects in red. Edge weights are proportional to the strength of standardized regression coefficients.

Our SEM detected a significant positive effect of vegetation cover (NLCD categories of deciduous trees, grassland, pasture, shrub) on diversity (estimate = 1.464 ± 0.299, z = 4.894, p < 0.001; Fig 5) and a significant negative effect of developed land (NLCD categories of low, medium, and high intensity developed land cover) on diversity (estimate = −0.207 ± 0.072, z = −2.888, p = 0.004). We did not find a significant direct effect of developed open space (z = 1.341, p = 0.180) on diversity (Fig 5). Significant negative covariance was observed between vegetation cover and development intensity (covariance = −0.014, z = −11.130, p < 0.001), and significant positive covariance between vegetation cover and open space (covariance = 0.002, z = 4.250, p < 0.001). Similarly, we found a significant negative covariance between development (high, medium, low intensity) and developed open space (covariance = −0.015, z = −12.100, p < 0.001). We also observed weak but significant positive covariance between developed low-intensity and developed open space (covariance = 0.005, z = 8.801, p < 0.001), and between grassland and shrub (covariance<0.001, z = 9.431, p < 0.001). We observed weak but significant negative covariance between developed high-intensity and developed low-intensity (covariance = −0.017, z = −11.109, p < 0.001), and deciduous and grassland (covariance = −0.001, z = − 3.218, p = 0.001). Further inspection of the structural relationships indicates that medium-intensity developed land is the primary driver of the negative direct effect of development on diversity, whereas high- and low-intensity development influence diversity more indirectly through their strong covariances with open space and deciduous tree cover. Among vegetation types, deciduous forest cover remains the only variable exerting a clear, positive direct effect on diversity, while grassland, shrubland, and pasture contribute indirectly through their positive associations with each other and with developed land types. The model also highlights significant negative covariances between developed and vegetated land covers, suggesting that increases in developed area are linked to concurrent reductions in vegetative habitat complexity..

## Discussion

Urbanization drastically alters landscapes, replacing naturally occurring habitats and vegetation with impervious surfaces and buildings. The process of urbanization not only impacts land cover type but also produces increased artificial light at night (ALAN), both of which can have significant consequences for biodiversity. Lepidoptera (moths and butterflies) are highly sensitive to land cover changes [29,56]. In this study, we examined how urbanization relates to the diversity of nocturnal Lepidoptera (moths), which perform vital ecosystem services and act as bioindicators [27]. Our findings suggest that urbanization is associated with lower moth diversity, with specific elements of the urban landscape shaping patterns of biodiversity in complex ways.

We found comparable moth community composition between locations with similar environments in both *iNaturalist* and our on-the-ground sampling datasets, and a general trend of decreasing diversity with increasing urbanization. The *iNaturalist* MRM had a low R² but was highly significant, likely reflecting high spatial coverage but heterogeneous observer effort. The on-the-ground MRM explained much more variation in community composition despite its smaller sample size and its limited spatial and temporal scope, suggesting that controlled sampling better captured ecological patterns among sites. These results suggest that moth community composition across New York City is strongly influenced by the environmental features that define different locations. Our linear models provide some insight into those features: the slopes of the relationships between moth biodiversity and medium-intensity urbanization were strongly negative, whereas those between diversity and deciduous tree cover and open land were strongly positive. In addition, our on-the-ground sampling found a strong negative relationship between moth diversity and light at night, with a high explained variance (R²), suggesting light at night accounts for a substantial portion of the observed variation in species diversity across sampled locations. Although several studies have found no consistent effects of light at night on biodiversity [10,57], our results are in line with those that identify significant impacts on moth community composition [58–60]. These negative effects on biodiversity are likely attributed to how light at night disrupts moth feeding, reproduction, migration, and development [20]. Alternatively, the lower biodiversity could simply reflect competition with other light sources in more heavily urbanized locations, resulting in an underestimation of the true moth diversity in heavily lit regions [61].

Our PCAs of *iNaturalist* and on-the-ground data reinforce these findings. When analyzed with linear regression, we found a strong negative slope describing the relationship between diversity and PC1 (primarily defined by high- and medium-intensity development), and a strong positive slope describing the relationship with PC3 (primarily deciduous tree cover and water). The repeated emergence of deciduous tree cover, open space, and high- and medium-intensity developed land as key variables across multiple analyses highlights the importance of these features. Our results are consistent with those of similar studies performed in metropolitan areas where highly disturbed habitats have been shown to support reduced moth species richness [19,22,62].

While we did not perform a SEM using our on-the-ground data due to the larger sample sizes required, our SEM using *iNaturalist* data and remotely sensed land cover found, similar to our other analyses, that higher diversity is associated with greater vegetation cover and less developed land. Further inspection of these relationships revealed that although vegetation and development have direct impacts on diversity, multiple indirect effects exist. Our strongest observed path within the SEM was the direct positive effect of multiple types of vegetation (deciduous trees, grassland, pasture, shrub) on diversity while development (high and medium intensity developed land) showed a weaker but significant negative direct effect. Indeed, the strong negative covariance between vegetation and development and a strong positive covariance between vegetation and developed open land, suggests that characteristics of more intense urbanization may indirectly drive reduced moth diversity by altering land cover compositions. Taken together, these results indicate that high- and medium-intensity development reduce moth diversity not only directly, but also indirectly through their association with reduced vegetation cover. Together, these results suggest that developed land conversion drives species declines independently and through strong indirect effects. This finding points to potential avenues for moth conservation through the maintenance of tree cover, vegetation, and open space even in highly developed parts of the city. Therefore, urban planning efforts focused on restoring parks and increasing vegetation across the city may make habitat more suitable for local moth populations.

Surprisingly, we did not find a relationship between air pollution and moth diversity at our on-the-ground sampling study sites. This finding contradicts our expectations, as researchers have documented declines across various taxa in response to higher levels of air pollution [62–63]. However, others suggest that the negative effects of air pollution on insects may have been overestimated [64]. It is also possible that our findings were influenced by confounding variables such as temperature and wind speed, as higher levels of particulate matter (PM2.5) coincided with warmer temperatures and lower wind speeds during sampling. A longer temporal scale may be required to detect a significant relationship between air pollution and moth biodiversity, given the high variation and sensitive nature of this variable. These factors highlight the complexity of isolating individual environmental drivers of diversity decline and underscore several constraints of our study.

We also acknowledge the inherent limitations of using *iNaturalist* data, which can be influenced by biases in socioeconomic factors and uneven sampling efforts. Research suggests that areas with higher socioeconomic status often experience greater sampling efforts by community scientists [65]. Taxonomic biases also exist; charismatic fauna, such as butterflies, are reported more frequently despite the greater overall diversity of moth species [66]. Both of these sources of bias are likely present in our dataset and may explain the overrepresentation of charismatic taxa such as Noctuidae and Erebidae. We also acknowledge that *iNaturalist* data might have misidentifications even among the Research Grade observations, and particularly for species that require dissection to definitively diagnose species. However, this is a common caveat of using large community-sourced datasets. Because of the potential impact of misidentification at the species level, we repeated all analyses at the genus level, finding highly concordant results for all analyses (S1 Appendix).

For these reasons we conducted on the ground surveys to reinforce our *iNaturalist* analyses. We also note that different species are active at different times of the night and throughout the season, thus the *iNaturalist* observations are expected to be more inclusive than our temporally restricted on-the-ground sampling. While our field data overall reiterated the same findings, it also further highlighted the limitations of citizen science data that emerged despite the greater sampling effort and temporal span. For example, our field sampling documented high diversity in Forest and Highland Parks, even though these parks have few moth records on *iNaturalist*. In fact, our on-the-ground sampling documented more moth species over a single evening at three locations in each of these parks than have ever been documented on *iNaturalist* for the entire park. Forest Park had 29 records on *iNaturalist* in over 20 years of observations in New York City, versus our 35 observations from our on-the-ground surveys; Highland Park is similar with 9 *iNaturalist* observations versus our 19. This contrast in species detection highlights the importance of targeted on-the-ground sampling to detect species; reliance on community-science data alone may underestimate true diversity. Indeed, we detected members of a family (Eriocraniidae) that had never been observed in New York City on *iNaturalist*, perhaps because of their particularly small size (~10 mm wingspan) and early spring emergence. However, the *iNaturalist* data recorded 40 families not observed in person. Moreover, *iNaturalist* has many records of larval stage moths, which would not be recorded during a light trap survey, and other adult stages of certain species which are difficult to record via light trapping due to variation in phototaxis [36]. The discrepancies between datasets highlight the importance of integrating *iNaturalist* data with on-the-ground field surveys to develop a more comprehensive understanding of biodiversity in a given area, and particularly in cities where habitat is highly heterogeneous and strong sampling biases can arise.

In conclusion, our analyses revealed significant relationships between moth diversity and urbanization at both landscape and local spatial scales. Our landscape-level analysis found that higher vegetation cover and developed open space support greater moth diversity, while our on-the-ground sampling revealed negative effects of light at night. Further, our findings highlight the importance of using advanced modeling approaches, such as SEM, alongside linear regression to reveal hidden or unobserved relationships in environmental data. Understanding these relationships is essential for designing targeted conservation strategies, as singular actions such as increasing vegetation alone, may be insufficient. We hope this study sparks further research into urban moth diversity, particularly given the essential role these organisms serve in the world’s ecosystems.

## Supporting information

S1 Table Summary of sampled locations for field sampling effort. (PDF)

S1 Appendix Analyses repeated at the genus level. (PDF)

S1 Fig Sampling density (number of records in a hex bin for *iNaturalist* sampling). (PDF)
