# Supplementary material for: From concrete to canopy: Illuminating moth biodiversity in New York City’s urban jungle
Source: PLoS One. 2026 May 12;21(5):e0342856. doi: 10.1371/journal.pone.0342856 (PMC13166911; doi:10.1371/journal.pone.0342856)
Supplement: S1 Table — (PDF) [file pone.0342856.s001.pdf]

Supplementary Materials
From Concrete to Canopy: Illuminating Moth Biodiversity in New York City's Urban Jungle

S1 Table. Summary of sampled locations for field sampling effort. Longitude and latitude, Simpson's D (calculated at the species-level), date sampled, and moon phase when sampled. Locations a, b, c at the larger parks were in different portions of the park.

Site	Longitude	Latitude	Species	Genera	Families	1-D	Date	Moon Phase
**Highland Park (a)**	40.6839918	-73.892613	6	5	4	0.81	3/12/24	Waxing crescent
**Highland Park (b)**	40.68483	-73.8879	2	2	2	0.50	4/8/24	New Moon
**Highland Park (c)**	40.6899	-73.889245	7	6	4	0.84	4/16/24	Waning Gibbous
**Forest Park (a)**	40.70065	-73.85007	4	4	3	0.72	3/15/24	Waxing crescent
**Forest Park (b)**	40.70439	-73.84373	11	10	6	0.88	4/29/24	Waning Gibbous
**Forest Park (c)**	40.707256	-73.849122	5	4	2	0.75	4/15/24	First Quarter
**Prospect Park (a)**	40.66367	-73.96464	4	3	3	0.72	3/3/24	Last Quarter
**Prospect Park (b)**	40.65827	-73.97208	2	2	2	0.44	4/9/24	Waxing crescent
**Prospect Park (c)**	40.66643	-73.96897	4	4	3	0.75	3/31/24	Waning Gibbous
**Maria Hernandez**	40.7034207	-73.924101	0	0	0	0	3/14/24	Waxing crescent
**Irving Square**	40.692754	-73.908968	0	0	0	0	4/10/24	Waxing crescent
**Grover Cleveland**	40.71127	-73.91157	3	3	3	0.67	4/28/24	Waning Gibbous
