# Supplementary material for: From concrete to canopy: Illuminating moth biodiversity in New York City’s urban jungle
Source: PLoS One. 2026 May 12;21(5):e0342856. doi: 10.1371/journal.pone.0342856 (PMC13166911; doi:10.1371/journal.pone.0342856)
Supplement: S1 Appendix — (PDF) [file pone.0342856.s002.pdf]

Supplementary Materials

From Concrete to Canopy: Illuminating Moth Biodiversity in New York City's Urban Jungle

S1 Appendix. Analyses repeated at the genus level.

S1.1 Comparison of Simpson's D calculated at the species and genus levels for field sampled data (A) and *iNaturalist* data (B).

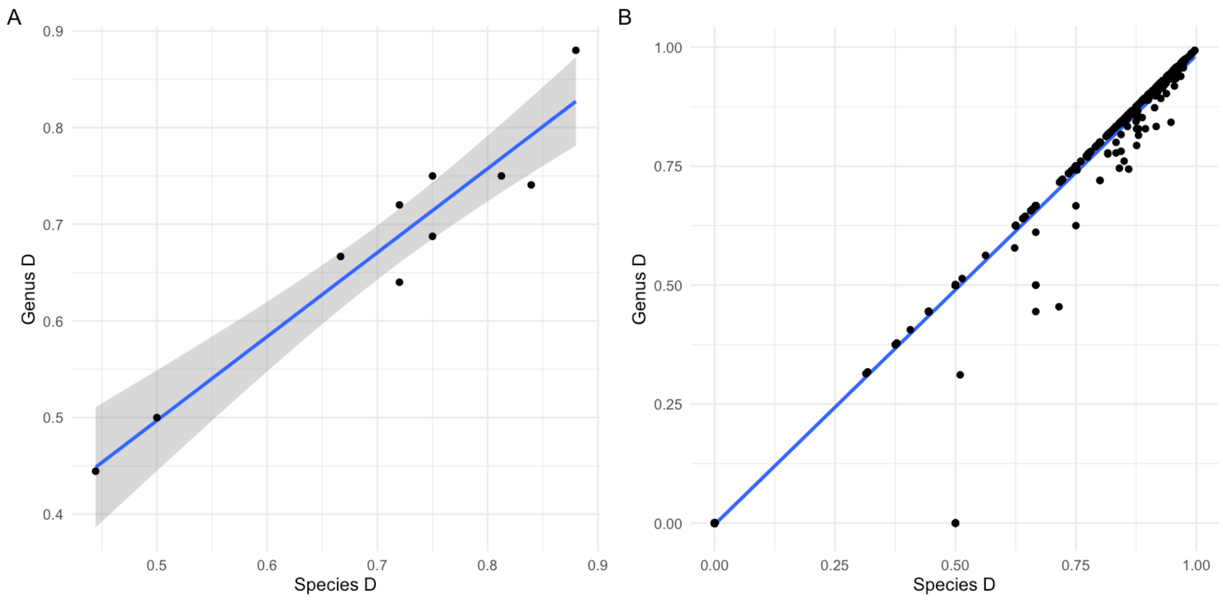

S1.2 Land cover linear model output for field survey data repeated at the genus level. The best fitting model (after model selection) again showed light at night as the only significant predictor variable. These results are almost identical to the analyses that use species-level diversity.

Model summary:

```
Call:
lm(formula = D ~ Pollution._PM25 + Lux, data = joined_data_genus)

Residuals:
     Min       1Q   Median       3Q      Max 
-0.23702 -0.03996  0.02674  0.05466  0.11050 

Coefficients:
                 Estimate Std. Error t value Pr(>|t|)    
(Intercept)      0.689612   0.055572  12.409 5.78e-07 ***
Pollution._PM25  0.008172   0.006062   1.348    0.211    
Lux             -0.482693   0.056492  -8.544 1.30e-05 ***
---
Signif. codes:  0 ‘***’ 0.001 ‘**’ 0.01 ‘*’ 0.05 ‘.’ 0.1 ‘ ’ 1

Residual standard error: 0.1012 on 9 degrees of freedom
Multiple R-squared:  0.8989,	Adjusted R-squared:  0.8764 
F-statistic: 39.99 on 2 and 9 DF,  p-value: 3.328e-05
```

S1.3 Land cover linear model output for *iNaturalist* data repeated at the genus level. We find the same five significant variables as the species-level model in the genus-level best model after AIC stepwise model selection: medium-intensity developed land, low-intensity developed land, developed open land, shrubland, and deciduous tree cover. Similar to the species diversity model, diversity was negatively associated with medium-intensity developed land cover and positively associated with deciduous tree cover and developed open space. There was again also a near-significant positive trend between diversity and low-intensity developed space, and although shrubland was retained in the best model, the relationship with this predictor was non-significant. These results are concordant with the species-level model.

Model summary:

```
Call:
lm(formula = D ~ Deciduous + Shrub + Developed_Medium_Intensity +
    Developed_Low_Intensity + Developed_Open_Space, data = div_results_genus)

Residuals:
     Min       1Q   Median       3Q      Max
-0.82948 -0.33182  0.06434  0.28398  0.74384

Coefficients:
                           Estimate Std. Error t value Pr(>|t|)
(Intercept)                 0.46452    0.02863  16.223  < 2e-16 ***
Deciduous                   0.46943    0.10697   4.388 1.31e-05 ***
Shrub                       1.68331    1.04139   1.616  0.10643
Developed_Medium_Intensity -0.33614    0.06365  -5.281 1.68e-07 ***
Developed_Low_Intensity     0.28358    0.15263   1.858  0.06356 .
Developed_Open_Space        0.35286    0.11839   2.980  0.00297 **
---
Signif. codes:  0 ‘***’ 0.001 ‘**’ 0.01 ‘*’ 0.05 ‘.’ 0.1 ‘ ’ 1

Residual standard error: 0.3453 on 750 degrees of freedom
Multiple R-squared:  0.139,	Adjusted R-squared:  0.1333
F-statistic: 24.23 on 5 and 750 DF,  p-value: < 2.2e-16
```

S1.4 Land cover linear model output for combined datasets repeated at the genus level. The relationships of genus diversity and the environmental principal components were highly similar to the species level model: in both diversity has a strong negative relationship with PC1 and a strong positive relationship with PC3, with no significant relationship to PC2 or PC4.

Model summary:

```
Call:
lm(formula = D ~ PC1 + PC2 + PC3 + PC4, data = merged_div3_genus)

Residuals:
     Min       1Q   Median       3Q      Max 
-0.70711 -0.32595  0.07409  0.31207  0.67237 

Coefficients:
             Estimate Std. Error t value Pr(>|t|)    
(Intercept)  0.434373   0.012691  34.226  < 2e-16 ***
PC1         -0.064327   0.008083  -7.958 6.30e-15 ***
PC2          0.003376   0.009506   0.355   0.7226    
PC3          0.046652   0.010684   4.367 1.44e-05 ***
PC4         -0.027046   0.015159  -1.784   0.0748 .  
---
Signif. codes:  0 ‘***’ 0.001 ‘**’ 0.01 ‘*’ 0.05 ‘.’ 0.1 ‘ ’ 1

Residual standard error: 0.3517 on 763 degrees of freedom
  (1 observation deleted due to missingness)
Multiple R-squared:  0.101,	Adjusted R-squared:  0.09633 
F-statistic: 21.44 on 4 and 763 DF,  p-value: < 2.2e-16
```

S1.5 Multiple matrix regression (MRM) repeated for genus-level data for field survey data. The MRM for the on the ground analyses using genus diversity was concordant with species-level data: locations with similar environments had similar community composition at the genus level.

Summary output:

```
$coef
                      lower(genus_dist_matrix)    pval
Int                                 3.19422969 0.88189
lower(genus_env_dist)               0.05760329 0.02467

$r.squared
       R2      pval
0.3008092 0.0246700

$F.test
       F   F.pval
18.49966  0.02467
```

S1.6 Multiple matrix regression (MRM) repeated for genus-level data for *iNaturalist* data. The MRM for the *iNaturalist* data at the genus level was also concordant with the analysis at the species level.

Summary output:

```
$coef
                        lower(g_inatdist_matrix)  pval
Int                                     3.617033 1e+00
lower(g_inatenv_matrix)                 3.989064 1e-05

$r.squared
       R2      pval
0.0133085 0.0000100

$F.test
         F     F.pval
3849.31435    0.00001
```

S1.7 SEM repeated at the genus level. The SEM using genus-level diversity produced results nearly identical to the species-level model. The overall fit of the model did not change across test statistics. Natural vegetation (x1) remained a strong positive predictor of diversity and developed land cover classes (x2) continued to show a significant negative effect. Open space (x3) again showed a small, non-significant positive relationship. Latent variable loadings and covariance structure were similar to the original model, indicating that the ecological signal persists regardless of whether diversity is measured at the species or genus scale. Overall, the genus-based SEM supports the same interpretation as the species-based model: vegetation promotes diversity while urban development suppresses it, with open space playing a weaker role.

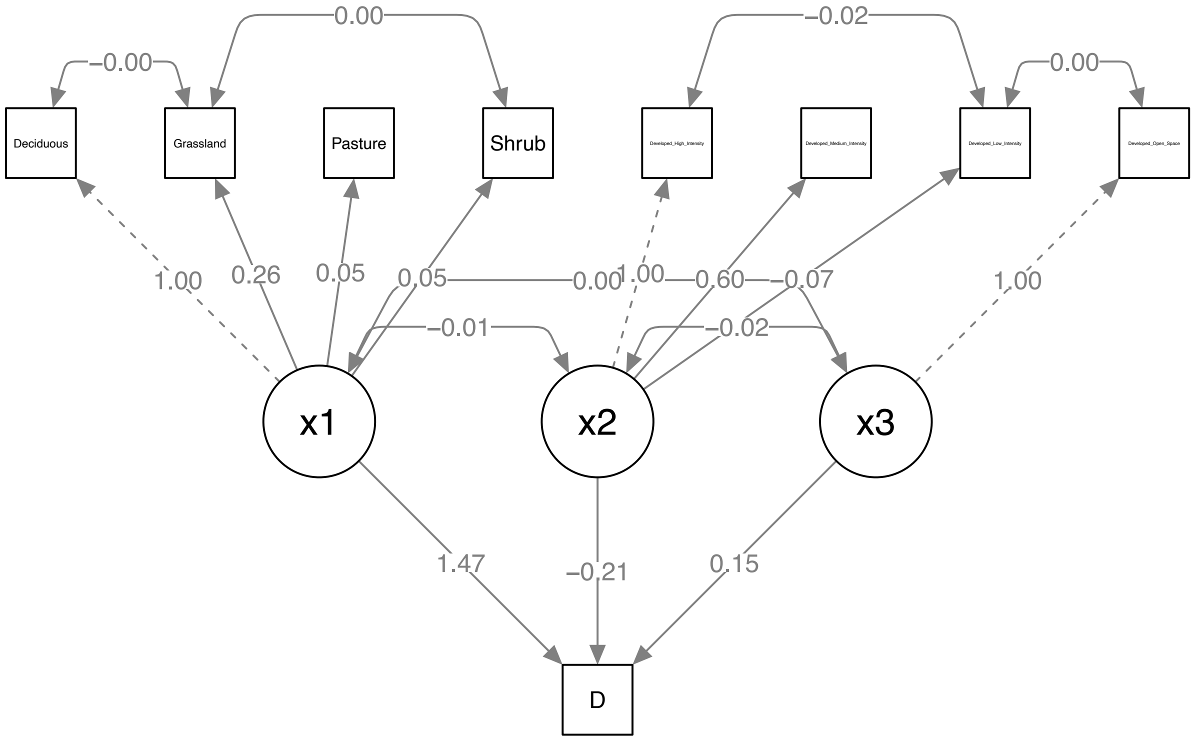

Model summary:

```
lavaan 0.6-19 ended normally after 541 iterations

  Estimator                                         ML
  Optimization method                           NLMINB
  Number of model parameters                        26

                                                  Used       Total
  Number of observations                           756         757

Model Test User Model:
  Test statistic                                83.710
  Degrees of freedom                                19
  P-value (Chi-square)                           0.000

Parameter Estimates:
  Standard errors                             Standard
  Information                                 Expected
```

```
  Information saturated (h1) model          Structured

Latent Variables:
                   Estimate  Std.Err  z-value  P(>|z|)   Std.lv  Std.all
  x1 =~
    Deciduous         1.000                               0.067    0.495
    Grassland         0.263    0.030    8.887    0.000    0.018    0.330
    Pasture           0.055    0.010    5.278    0.000    0.004    0.170
    Shrub             0.051    0.008    6.638    0.000    0.003    0.219
  x2 =~
    Dvlpd_Hgh_Intn    1.000                                  NA       NA
    Dvlpd_Mdm_Intn    0.605    0.071    8.567    0.000       NA       NA
    Dvlpd_Lw_Intns   -0.071    0.020   -3.477    0.001       NA       NA
  x3 =~
    Devlpd_Opn_Spc    1.000                               0.132    1.000

Regressions:
                   Estimate  Std.Err  z-value  P(>|z|)   Std.lv  Std.all
  D ~
    x1                1.472    0.299    4.921    0.000    0.099    0.267
    x2               -0.207    0.072   -2.885    0.004       NA       NA
    x3                0.155    0.130    1.186    0.236    0.020    0.055

Covariances:
                               Estimate  Std.Err  z-value  P(>|z|)   Std.lv  Std.all
  x1 ~~
    x2                           -0.014    0.001  -11.124    0.000   -1.439   -1.439
    x3                            0.002    0.001    4.240    0.000    0.256    0.256
  x2 ~~
    x3                           -0.015    0.001  -12.101    0.000   -0.811   -0.811
 .Developed_Low_Intensity ~~
   .Devlpd_Opn_Spc                0.005    0.001    8.807    0.000    0.005      Inf
 .Developed_High_Intensity ~~
   .Dvlpd_Lw_Intns               -0.017    0.002  -11.108    0.000   -0.017   -0.545
 .Grassland ~~
   .Shrub                         0.000    0.000    9.423    0.000    0.000    0.403
 .Deciduous ~~
   .Grassland                    -0.001    0.000   -3.219    0.001   -0.001   -0.139

Variances:
                   Estimate  Std.Err  z-value  P(>|z|)   Std.lv  Std.all
   .Deciduous         0.014    0.001   12.489    0.000    0.014    0.755
   .Grassland         0.003    0.000   17.616    0.000    0.003    0.891
   .Pasture           0.000    0.000   19.405    0.000    0.000    0.971
   .Shrub             0.000    0.000   19.221    0.000    0.000    0.952
   .Dvlpd_Hgh_Intn    0.098    0.007   13.244    0.000    0.098    1.257
   .Dvlpd_Mdm_Intn    0.055    0.003   15.915    0.000    0.055    1.153
   .Dvlpd_Lw_Intns    0.010    0.001   19.251    0.000    0.010    1.010
   .Devlpd_Opn_Spc    0.000                               0.000    0.000
   .D                 0.118    0.007   16.167    0.000    0.118    0.857
    x1                0.005    0.001    4.280    0.000    1.000    1.000
    x2               -0.020    0.004   -4.471    0.000       NA       NA
    x3                0.017    0.001   19.860    0.000    1.000    1.000
```
