# Supplementary material for: From concrete to canopy: Illuminating moth biodiversity in New York City’s urban jungle
Source: PLoS One. 2026 May 12;21(5):e0342856. doi: 10.1371/journal.pone.0342856 (PMC13166911; doi:10.1371/journal.pone.0342856)

Supplementary Materials

From Concrete to Canopy: Illuminating Moth Biodiversity in New York City's Urban Jungle

S1 Fig. Sampling density (number of records in a hex bin for *iNaturalist* sampling). (A) Histogram of samples per hex bin, with mean number of records = 12.4 (red vertical line) and few hex cells containing larger numbers of records. (B) Number of records per hex bin versus Simpson's D illustrating high values of D are attained even at low sampling densities.

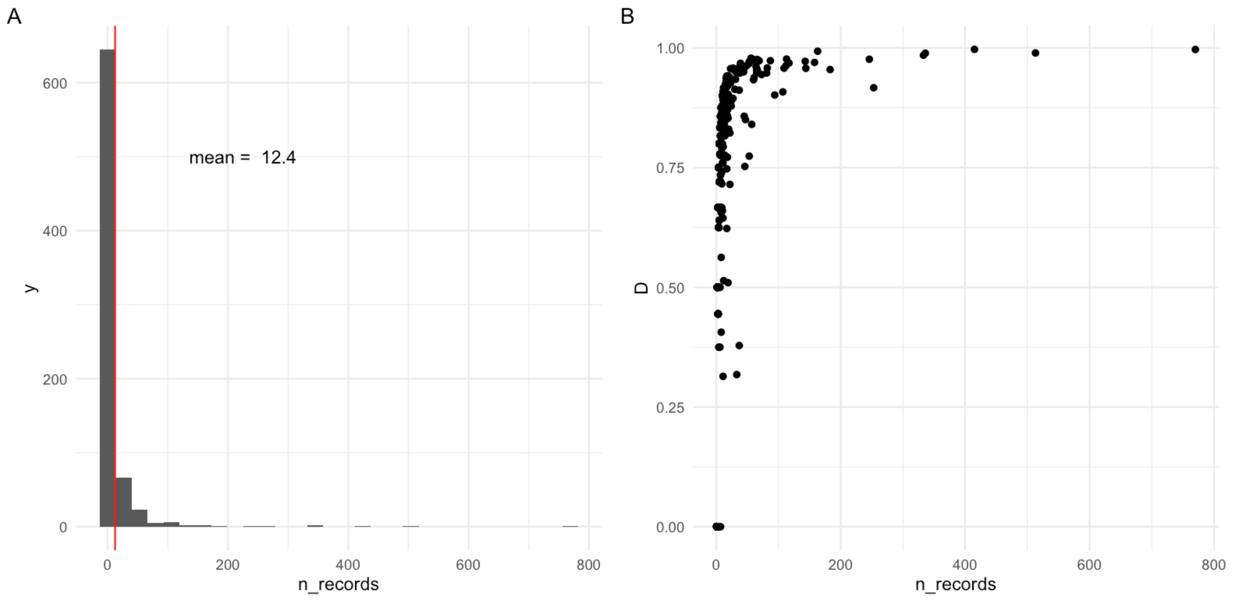

Supplement: S1 Fig — (PDF) [file pone.0342856.s003.pdf]
